# Laryngeal involvement causing dysphonia in a 29 year old nursing mother with lepromatous leprosy

**DOI:** 10.11604/pamj.2015.21.146.7098

**Published:** 2015-06-23

**Authors:** Sombo Fwoloshi, Sharon Musonda Machona, Victor Mudenda, Owen Ngalamika

**Affiliations:** 1 Infectious Diseases Unit, Department of Medicine, University Teaching Hospital, Lusaka, Zambia; 2 Dermatovenereology Section, Department of Medicine, University Teaching Hospital, Lusaka, Zambia; 3 Department of Pathology, University Teaching Hospital, Lusaka, Zambia

**Keywords:** Leprosy, atypical features, dysphonia, deep cutaneous mycosis

## Abstract

Leprosy is a granulomatous disease that mainly affects the skin and peripheral nerves. It is caused by infection with *mycobacterium leprae* or *mycobacterium lepromatosus*. In most instances, diagnosis of leprosy can easily be made based on the clinical signs and symptoms. However, when patients present with atypical features, clinical diagnosis can be a challenge. We report a case of a nursing mother with lepromatous leprosy who presented with dysphonia and skin lesions initially thought to be a deep cutaneous mycosis.

## Introduction

Leprosy is a granulomatous disease caused by *mycobacterium leprae* and less frequently by *mycobacterium lepromatosus* [1]. A delay in treatment of leprosy is usually a consequence of delayed diagnosis rather than due to absence of recommended drugs. When typical cutaneous lesions and peripheral nerve involvement are the presenting features, diagnosis of leprosy is easy and early. However, diagnosis can be elusive when less common presentations such as laryngeal and ocular manifestations are encountered. Zambia is considered a low prevalence area for leprosy. Nevertheless, like some countries in Africa we continue to have several cases of the disease diagnosed every year. We report the case of a woman referred to us from an area with a leprosy center who had a pattern of disease not initially attributed to leprosy.

## Patient and observation

**Figure 1:**
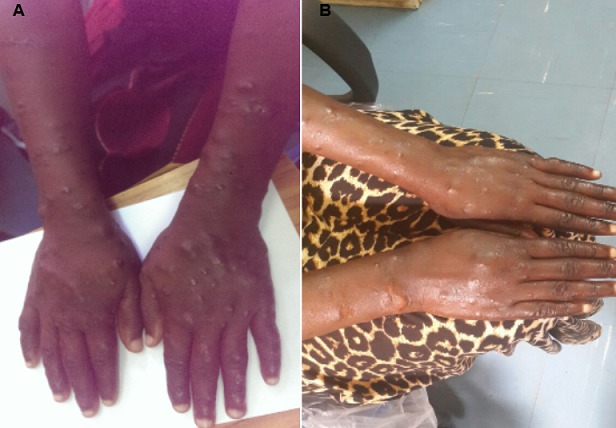
A) skin colored papules and nodules on dorsum of hands and posterior aspect of both forearms present at initial presentation of the patient; B) reduced number and size of skin lesions about a month after commencing multidrug treatment for multibacillary leprosy

**Figure 2:**
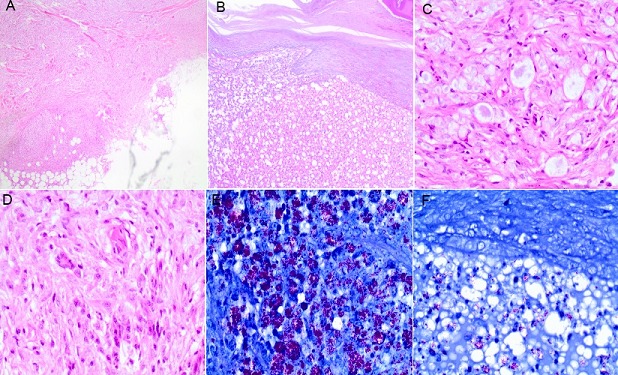
A) granulomas in dermis extending into fatty tissue; B) Normal epidermis, vacuolation and foam cells in dermis, chronic inflammatory cells also present (H&E, x40); C) Foam cells with bacilli (H&E, x400); D) granulomas with langerhan's giant cells (H&E, x400); E) Mirriads of bacilli (Wade-fite stain, x400); F) Lower epidermis, and cells with multiple bacilli in the upper dermis (Wade-fite, x400)

A 29 year old woman was referred to the university teaching hospital (UTH) from a central hospital for further evaluation and management of generalized skin lesions which had been present for several years. The skin lesions were initially thought to be psoriasis or syphilis. They had started off as painful growths under the skin which would eventually discharge mucoid fluid. Over the past 2 to 3 months prior to presentation at UTH, she experienced an increase in the number and size of skin lesions with involvement of previously unaffected areas like the face. She also developed a persistent sore throat with associated dysphonia and occasional fevers. Furthermore, she reported blurring of vision and gritty sensation in the left eye. Her husband and 3 children were all well. She noted exacerbation of her condition during her last pregnancy for which she had delivered about 6 months before presentation. On physical examination, generalized multiple papules and nodules concentrated over the extremities were seen (Figure 1 (A)). Few nodules were also present on facial skin, with loss of eyebrow density on the right side. In addition, some erythematous papules were noted on the posterior aspect of her tongue. She underwent an ophthalmologist exam where the left eye was found to be injected and thought to be an infected pinguecula. A nasal examination by the otolaryngologist revealed grey crusts in the nasal mucosa and laryngoscopy revealed oedema of the epiglottis and false vocal cords. Her neurological examination was non-revealing and no nerves were palpable. Her 6 month old infant whom she had come with was in good health. An initial diagnosis of disseminated deep cutanous mycosis to rule out leprosy was made. A wedge skin biopsy taken from her left arm underwent histopathological evaluation. PAS-staining for fungal elements was negative. Basic hematoxyllin and eosin staining revealed multiple granulomas in the dermis. On further staining with wade-fite, multiple bacilli were seen (Figure 2). This was followed by ZN-stained slit skin smear microscopy which showed numerous acid-fast bacilli. Results of other investigations done are shown in the table below (Table 1). A final diagnosis of multibacillary leprosy was made. Treatment with dapsone, rifampicin, and clofazimine was commenced with a plan to follow up her infant for the next five years. She returned for a review about a month after commencing treatment and her lesions had reduced in size and number (Figure 1 (B)), with normalization of her voice.

**Table 1:** Results of ordered investigations

Test	Result	Reference Range
White cell count	3.59x10^∧^9/l	4.00-10.00x10^∧^9/l
Haemoglobin	11.6g/dl	12.1-16.3g/dl
Liver function tests	normal	
Renal function tests	Normal	
Chest X-ray	Clear lung fields	
Skin Biopsy	Histopathological features of leprosy	
HIV	Negative	

## Discussion

This case highlights some atypical and misleading presentations of leprosy. Classical features of leprosy are dependent on the clinical subtype and may include peripheral nerve enlargement and characteristic anaesthetic cutaneous lesions [2]. However, a handful of patients may have an atypical presentation which may not readily be recognized as leprosy. This patient had skin lesions that may be encountered in deep cutaneous mycoses like disseminated cutaneous sporotrichosis [3]. Other atypical presentations reported in the literature include ocular (keratitis, uveitis) [4] and upper airway involvement (laryngitis) [2]. Leprosy has been classified into 2 polar forms (lepromatous and tuberculoid) with 3 borderline disease patterns in between [5]. This is a result of a genetically predetermined immune response in different individuals [6]. The WHO classifies it into paucibacillary and multibacillary depending on the burden of bacilli. Our patient had lepromatous leprosy which is consistent with multibacillary leprosy and evident from the slit smear. The patient initially denied anyone in her family having a similar disease. However, upon confirming the diagnosis and further questioning, she mentioned that her father had undergone treatment for leprosy from the referring hospital and that three of her uncles from her father's side were also treated for leprosy several years ago. This may be explained by possible individual differences in immune responses leading to different clinical subtypes of leprosy and atypical presentations such as laryngitis causing dysphonia. Involvement of the larynx requires early treatment and close observation as it may result in upper airway obstruction [7]. Humans and armadillos are the only known reservoirs of leprosy. The bacilli thrive at cool temperatures of 27 to 33°C hence abundance of lesions on the extremities, face, and ears. The BCG vaccine which was initially developed to provide protection against tuberculosis also protects against leprosy. Furthermore, chemoprophylaxis with rifampicin has been shown to be effective [8] and is widely used in countries still battling high incidence rates of leprosy. Exposed infants are usually asymptomatic, but this is the period of acquisition for childhood and adolescent leprosy, hence the need to follow up the infant and his siblings. However, whether they become symptomatic and the clinical subtype will be determined by heritable factors.

## Conclusion

Most cases of lepromatous leprosy are clinically obvious. However, when it presents in an unusual manner, histopathology is a very valuable tool in distinguishing it from other disease entities. It is vital to diagnose and commence treatment early in patients presenting with laryngeal involvement so as to prevent fatal complications such as upper airway obstruction in leprosy patients.
